# Glancing at the major issues of the Animal Bioscience Forum 2020

**DOI:** 10.5713/ab.2021.0002ED

**Published:** 2021-04-22

**Authors:** Cheol-Heui Yun

**Affiliations:** 1 Department of Agricultural Biotechnology and Research Institute of Agriculture and Life Sciences, Seoul National University, Seoul 08826, Korea; 2 Co Editor-in-Chief, Animal Bioscience, Seoul 08776, Korea

Since early 2020, we have experienced a worldwide economic downturn and depression because of the COVID-19 pandemic in each and every industry, including the production of economic animals and their valued goods. It is certain that the global population, and consequently the demand for animal-derived protein-rich foods, will continue increasing in the next 30 years; however, the sufficiency of these supplies is uncertain. In the fields of animal science and biotechnology, in order to meet the requirements of our fast-changing world, the Animal Bioscience (AB, formerly called Asian-Australasian Journal of Animal Sciences) held the Animal Bioscience Forum 2020 which focused on animal bioscience for the improvement of animal health and production (December 9 to 10, 2020). Four speakers were invited to a virtual live online forum on various subjects in the fields of immunology, herb, and system of monogastric animals. Video versions of all presentations are available on the homepage of the AB (https://www.animbiosci.org/index.php?v_type=7). Accordingly, a Special Issue including 12 peer-reviewed articles was published (https://www.animbiosci.org/index.php?v_type=5&year=2020), on which the topics closely related with the theme of the Animal Bioscience Forum 2020.

In the first review article, entitled ‘Immunosecurity: Immunomodulants enhance immune responses in chickens’, Yu et al [1] focused on the stressors that can negatively affect the immune system of chickens (imbalanced nutrition and dysbiosis). The authors introduced various immunomodulators to alleviate stress and tension, induce changes in the intestinal microbiota to obtain more favorable conditions, and enhance protective immune responses in chickens. From this article, the readers will gain comprehensive knowledge about the relationship between stress and immune responses in chickens in relation to the use and impact of immunomodulators that can potentially replace antimicrobial growth factors.

The second article, entitled ‘Understanding intestinal health in nursery pigs and the relevant nutritional strategies’, by Kim and Duarte [2] discussed how piglets weaned at an early age with immature intestine (jejunum in particular) respond to dramatic changes in dietary and environmental factors. The study explained the challenges of maintaining a healthy status and growth potential of nursery piglets and defining biomarkers. The authors emphasized the importance of mucosa-associated microbiota in the jejunum related to the use of selected prebiotics, probiotics, postbiotics, and other bioactive compounds.

The third article, entitled ‘Phytobiotics to improve health and production of broiler chickens: functions beyond the antioxidant activity’ and authored by Kikusato [3], introduced various biological activities of phytobiotics as alternatives to synthetic antibiotic growth promoters. Besides the traditionally known antioxidant activity of phytobiotics, the author described the results of numerous studies conducted on their anti-inflammatory, antimicrobial, and transcriptional activities at the molecular and cellular levels. The readers of this article will gain extensive knowledge about the bioavailability and potential action mechanism of phytobiotics underlying their effects on gastrointestinal physiology.

The fourth article, entitled ‘Precision feeding and precision nutrition: A paradigm shift in broiler feed formulation?’ by Moss et al [4], discussed the practical process and the frequency of diet administration using a new technology to blend feed and match the daily nutrient requirements of broilers. The authors introduced feed phases on nutrient utilization and growth performance along with precision nutrition and feeding the broilers a new diet every day in the production cycle. The readers of this article will gain insight into a new technology for precision nutrition regimens targeting daily requirements by efficiently blending dietary components with the aim to improve growth performance and reduce production costs, consequently increasing economic benefits and sustainability of the chicken industry.

The review articles introduced in this special issue will provide necessary information for producers and research scientists in the area of domestic animal bioscience who are looking for ideas on new ingredients and advanced technology for achieving better growth performance and at the same time maintaining the healthy status of the animals.
